# Rates of obstetric intervention during birth and selected maternal and perinatal outcomes for low risk women born in Australia compared to those born overseas

**DOI:** 10.1186/1471-2393-13-100

**Published:** 2013-05-01

**Authors:** Hannah G Dahlen, Virginia Schmied, Cindy-Lee Dennis, Charlene Thornton

**Affiliations:** 1 School of Nursing and Midwifery, University of Western Sydney, Locked Bag 1797, Penrith South DC, NSW, 2751, Australia; 2 Department of Psychiatry; Canada Research Chair in Perinatal Community Health; Shirley Brown Chair in Women's Mental Health Research, Women's College Research Institute, University of Toronto Lawrence S. Bloomberg Faculty of Nursing, 155 College St, Toronto, ON, M5T 1P8, Canada

**Keywords:** Migrant, Immigrant, Induction, Vaginal birth, Obstetric care, Caesarean, Multicultural, Indian, Lebanese

## Abstract

**Background:**

There are mixed reports in the literature about obstetric intervention and maternal and neonatal outcomes for migrant women born in resource rich countries. The aim of this study was to compare the risk profile, rates of obstetric intervention and selected maternal and perinatal outcomes for low risk women born in Australia compared to those born overseas.

**Method:**

A population-based descriptive study was undertaken in NSW of all singleton births recorded in the NSW Midwives Data Collection between 2000–2008 (n=691,738). Risk profile, obstetric intervention rates and selected maternal and perinatal outcomes were examined.

**Results:**

Women born in Australia were slightly younger (30 vs 31 years), less likely to be primiparous (41% vs 43%), three times more likely to smoke (18% vs 6%) and more likely to give birth in a private hospital (26% vs 18%) compared to women not born in Australia. Among the seven most common migrant groups to Australia, women born in Lebanon were the youngest, least likely to be primiparous and least likely to give birth in a private hospital. Hypertension was lowest amongst Vietnamese women (3%) and gestational diabetes highest amongst women born in China (14%). The highest caesarean section (31%), instrumental birth rates (16%) and episiotomy rates (32%) were seen in Indian women, along with the highest rates of babies <10th centile (22%) and <3rd centile (8%). Lebanese women had the highest rates of stillbirth (7.2/1000). Similar trends were found in the different migrant groups when only low risk women were included.

**Conclusion:**

The results suggest there are significant differences in risk profiles, obstetric intervention rates and maternal and neonatal outcomes between Australian-born and women born overseas and these differences are seen overall and in low risk populations. The finding that Indian women (the leading migrant group to Australia) have the lowest normal birth rate and high rates of low birth weight babies is concerning, and attention needs to be focused on why there are disparities in outcomes and on effective models of care that might improve outcomes for this population.

## Background

One in four Australians are born overseas with 44% either born overseas or having a parent who was. More than 270 ancestries are identified and 4 million people in Australia speak another language other than English, with over 260 languages spoken [1]. Australia’s multicultural composition has been described as at the heart of the national identity and intrinsic to the history and character of the nation [1]. The numbers of Australians born in Europe has declined over the past decade and those born in East, Central and Southern Asia has increased [1]. In 2012 India became the largest source of permanent migrants to Australia, surpassing China and the UK [2].

Birth is important to women of all cultural backgrounds, but meaning and understandings of birth may differ considerably, along with physical and psychological risk factors that can impact on pregnancy and birth outcomes. As ethnically diverse migrant women settle in resource rich countries, several social, psychological and biological factors need to be considered when providing care during the childbearing phase of their lives [3]. Studies have demonstrated non-white ethnicity and migrant status to be a predictor of severe maternal morbidity [4] and mortality [5] and that migrant women are more likely to be multiparous and have pregnancy-related diseases which can impact on health outcomes for themselves and their babies [3,6-9]. Perinatal and infant mortality has been shown to be higher in several studies for migrant women [3,10-12]. This has been attributed to several factors including an increased incidence of low birth weight babies [3]. Studies have indicated that sociodemographic factors, such as income, may have an influence on birth weight [13,14] and preterm birth [15] and these factors affect migrants to a greater extent. Low birth weight is associated with increased morbidity and mortality throughout the life course, such as impaired cognitive function, decreased insulin sensitivity and an increased risk of metabolic syndrome [13,16,17]. Other studies have found maternal education to be more strongly associated with low birth weight and preterm birth than income [15].

Birth interventions such as caesarean section have also been reported to be higher in certain migrant groups [3,9,12,18,19]. Whether women seek care during pregnancy or are able to understand health advice given to them is influenced by their concepts of health and illness, health literacy, knowledge of the health service and financial status [20,21]. Many different factors can impact on the quality of care experienced by migrant women, including communication problems due to language skills or health professional attitudes [22,23], clinical appropriateness [24] and use of services [25-27]. Other studies however have demonstrated good outcomes for immigrant women (the healthy migrant effect) despite the presence of demographic and socioeconomic risk factors [28]. Questions are also raised about appropriateness of standardised measures such as gestational age and birth weight for different races, with calls for ethnic-specific perinatal health indicators to be developed [3,29]. Finally, and perhaps most importantly, migrant women have identified in several studies the need for safe, kind, supportive care [5,30,31] with communication identified as a key factor in satisfaction with care.

The aim of this study was to determine the rates of obstetric intervention and selected maternal and neonatal outcomes for low risk women born in Australia compared to those born overseas giving birth in New South Wales (NSW) between 2000–2008.

## Methods

### Data sources

Perinatal data recorded in the NSW Midwives Data Collection (MDC) and the NSW Register for Congenital Conditions for the time period July 1st 2000 till June 30th 2008 was provided by NSW Department of Health. The MDC is a population-based surveillance system containing maternal and infant data on all births of greater than 400 grams birth weight or 20 weeks gestation. The NSW Register of Congenital Conditions (formerly the NSW Birth Defects Register) is a population-based surveillance system established to monitor congenital conditions detected during pregnancy or at birth, or diagnosed in infants up to one year of age.

The linked datasets were provided by the NSW Centre for Health Record Linkage (CHeReL) following approval by the Data Custodian (NSW Health).

Ethical approval was obtained from the NSW Population and Health Services Research Ethics Committee, Protocol No.2010/12/291.

### Outcome measures

Maternal factors available for analysis included: country of birth, age, parity, smoking status (any smoking during pregnancy), whether the mother gave birth in a private hospital, and pre-existing (pre-pregnancy diabetes and chronic hypertension) and pregnancy-related medical conditions (pregnancy-related diabetes and hypertensive disorders of pregnancy). Teenage pregnancy was defined by maternal age less than 20 years of age at time of delivery. Country of birth of the mother was determined utilizing the Australian Bureau of Statistics Standard Australian Classification of Countries [32]. Obstetric factors included labour onset, delivery type, pain relief utilised and perineal status. Labour onset was categorised as spontaneous or induced and/or augmented by means of prostaglandins, synthetic oxytocins and/or mechanical devices but not artificial rupture of membranes alone. Caesarean sections were divided into ‘elective’, those where the woman was recorded as having ‘no labour’ and ‘emergency’, regardless of spontaneous or induced/augmented labour onset. Neonatal factors included birth weight, gestation at birth, presentation and Apgar Scores. Neonatal deaths were calculated within 28 days of birth. Birth weight centiles were calculated from within the dataset and were adjusted for sex and gestation at birth. Birth weights < 10th centile (adjusted for sex and gestation) < 3rd centile (adjusted for sex and gestation) were used. Epidural use was calculated from two variables as the form was altered during the time period of the study and was limited to those women who used an epidural for pain relief in labour. Severe perineal trauma is a variable applied to any women coded as having a 3rd or 4th degree tear or any woman coded as having an episiotomy/tear combination. Separate variables were recoded to create tear/episiotomy combination. Episiotomy was also included as a variable. The denominator used was vaginal births only for perineal trauma, excluding caesarean sections.

The low risk primipara was defined as a primiparous woman aged 20–34 years, who had no pre-existing or pregnancy-related hypertension or diabetes, who gave birth to a singleton baby at 37–41 weeks gestation in a cephalic presentation within the 10th and 90th centiles for birth weight. The low risk multipara was a multiparous woman aged 20–34 years, who had no pre-existing or pregnancy-related hypertension or diabetes, who gave birth to a singleton baby at 37–41 weeks gestation in a cephalic presentation within the 10th and 90th centiles for birthweight. We excluded women who smoked during pregnancy as there is a strong association between poor pregnancy outcomes and smoking.

This model has been used in several papers in the past by ourselves and other authors [33,34]. We acknowledge that several different definitions have been used in studies in Australia and overseas for low risk cohorts. Babies with congenital conditions were removed from the analysis.

Stillbirths and neonatal deaths were calculated from the MDC following the removal of infants with congenital abnormalities as recorded in the NSW Register of Congenital Conditions.

### Data analysis

In order to examine differences in obstetric intervention rates and maternal and neonatal outcomes, all analyses were completed by comparing Australian-born women to women from the seven countries which had more than 1% of women giving birth in NSW during the period of 2000–2008 (New Zealand, England, China, Vietnam, Lebanon, Philippines, and India). We also compared Australian-born women to all other women, which comprised all the remaining women who were born in 257 countries other than Australia. Student t-tests, ANOVA and chi-square analyses were conducted as appropriate. A significance level of <0.01 was set due to the nature of population data and the significant size of the dataset. All analyses were conducted using IBM SPSS v.19®.

## Results

### Overall comparisons

Nearly 72% (n=496,668) of women in this study were born in Australia and 28% (195,070) were born in a country other than Australia. There were seven countries where more than one percent of the birthing women were represented. They are in order, New Zealand (2.5%), England (2.2%), China (2.1%), Vietnam (2.0%), Lebanon (1.8%), Philippines (1.4%) and India (1.2%). A further 15% of women were born in 257 countries other than Australia that each comprised less than one percent of the population giving birth (Table 1).

**Table 1:** Distribution of participants by country of birth

**Country of birth**	**N=691,738**	**% in cohort**
Australia	496668	71.8%
New Zealand	17293	2.5%
England	15218	2.2%
China	14526	2.1%
Vietnam	13835	2.0%
Lebanon	12451	1.8%
Philippines	9684	1.4%
India	8301	1.2%
All other countries	103761	15.0%

*Due to rounding this may not equal total.

Women born in Australia compared to women not born in Australia, were slightly younger (29.8 vs 31.4 years), more likely to be teenagers (<20 years of age at delivery) (5.1% vs 1.6%), less likely to be over 35 years of age (17.8% vs 26.2%) and less likely to be primiparous (41.2% vs 42.9%); however women born in some countries, such as India, were much more likely to be primiparous (Tables 2 &3). Australian-born women were three times more likely to smoke (18.3% vs 6.1%) and more likely to give birth in a private hospital (26% vs 17.7%); however, women born in NZ had the highest rates overall of smoking in pregnancy and women born in England were just as likely to give birth in a private hospital compared to Australian born women. Australian-born women were more likely to be diagnosed with pregnancy-related hypertension, have a caesarean section, have an epidural and a higher birthweight baby than women born in countries other than Australia. Australian-born women were however less likely to be diagnosed with gestational diabetes (3.1% vs 7.5%), to have an instrumental birth (10.1% vs. 11.7%), to have a baby with a birth weight <10th centile (9.4% vs. 11.9%) or < 3rd centile (2.8% vs. 3.5%). There was no difference between Australian-born and non-Australian-born women in severe perineal trauma rates. There are however variations in demographics and obstetric complications and outcomes between some of the individual ethnic groups and Australian born women (Table 3).

**Table 2:** Selected demographics, maternal obstetric history and maternal and perinatal outcomes among Australian-born and non-Australian-born women, N=691,738

	**Australian-born**	**Non-Australian-born**	**p**
	**n=496 668**	**n=195 070**	
Maternal age (mean SD)	29.8 (5.59)	31.4 (5.38)	<0.0001
Teenage pregnancy	5.1%	1.6%	<0.0001
Pregnancy ≥35 years	17.8%	26.2%	<0.0001
Primiparous	41.2%	42.9%	<0.0001
Smoking	18.3%	6.1%	<0.0001
Birth in private hospital	26.0%	17.7%	<0.0001
Hypertensive Disease of Pregnancy	6.0%	4.7%	<0.0001
Gestational Diabetes Mellitus	3.1%	7.5%	<0.0001
Gestation at delivery (mean SD)	39.1 (2.07)	39.09 (2.05)	<0.001
Spontaneous labour	68.6%	75.7%	<0.0001
Pre-term birth (<37 weeks)	6.0%	5.5%	<0.001
Normal vaginal delivery	63.3%	63.1%	<0.001
Assisted vaginal delivery	10.1%	11.7%	<0.0001
Caesarean section (elective)	15.2%	14.0%	<0.0001
Caesarean section (emergency)	11.3%	11.7%	<0.0001
Stillbirth Rate/1 000 births	5.2/1 000	5.3/1 000	0.08
Neonatal death Rate/1 000 livebirths	1.9/1000	2.0/1 000	0.23
Epidural usage	27.0%	25.2%	<0.0001
No pain relief for labour	8.1%	8.5%	<0.0001
Episiotomy#	14.3%	18.4%	<0.0001
Severe perineal trauma (all women)#	1.6%	1.5%	0.44
Severe perineal trauma (primips only)#	1.6%	1.6%	0.54
Birth weight (mean SD)	3423 (580.9)	3347 (560.6)	<0.0001
Birth weight <10th centile	9.4%	11.9%	<0.0001
Birth weight <3rd centile	2.8%	3.5%	<0.0001
5 minute Apgar <7	2.0%	2.0%	0.94

*Following removal of those with congenital abnormalities.

#For severe perineal trauma and episiotomy, caesarean sections were removed for analysis.

**Table 3:** Selected demographics, maternal obstetric history and maternal and perinatal outcomes among Australian-born women and women born in most common migrant groups, N=691,738

	**Australia**	**New Zealand**	**England**	**China**	**Vietnam**	**Lebanon**	**Philippines**	**India**	**Other**	**p**
	**n=496 668**	**n=17 293**	**n=15 218**	**n=14 527**	**n=13 835**	**n=12 451**	**n=9 684**	**n=8 301**	**n=103 761**	
Maternal age (mean SD)	29.8 (5.59)	30.2 (5.95)	33.8 (4.66)	32.8 (4.91)	30.9 (4.90)	29.3 (5.90)	31.2 (5.72)	30.1 (4.29)	31.5 (5.27)	<0.0001*
Teenage pregnancy	5.1%	2.4%	0.3%	0.2%	0.4%	1.5%	1.1%	0.1%	0.5%	<0.0001
Pregnancy ≥35 years	17.8%	22.8%	42.1%	33.8%	20.5%	18.4%	27.1%	13.2%	26.0%	<0.0001
Primiparous	41.2%	39.6%	45.6%	50.0%	43.2%	27.7%	40.5%	52.9%	43.3%	<0.0001
Smoking	18.3%	22.6%	8.3%	0.5%	1.3%	8.6%	3.9%	1.6%	4.8%	<0.0001
Birth in a private hospital	26.0%	14.0%	25.8%	13.1%	11.5%	8.4%	12.5%	18.5%	20.2%	<0.0001
Hypertensive Disease of Pregnancy	6.0%	6.5%	6.3%	2.8%	2.5%	3.1%	6.8%	4.6%	4.8%	<0.0001
Gestational Diabetes Mellitus	3.1%	3.3%	3.6%	13.8%	11.4%	6.4%	9.8%	9.6%	7.0%	<0.0001
Gestation at delivery (mean SD)	39.1 (2.06)	39.2 (2.12)	39.2 (1.97)	39.1 (1.90)	38.9 (2.04)	39.2 (2.07)	38.7 (2.09)	39.0 (2.05)	39.1 (2.06)	<0.0001*
Pre-term birth (<37 weeks)	6.0%	6.1%	4.8%	4.3%	6.0%	4.8%	7.1%	5.7%	5.5%	<0.0001
Spontaneous labour	58.2%	64.8%	58.1%	66.1%	75.4%	69.4%	69.4%	60.7%	64.1%	<0.0001
Normal vaginal delivery	62.9%	67.8%	58.7%	57.1%	69.0%	76.8%	58.9%	52.6%	61.9%	<0.0001
Assisted vaginal delivery	10.1%	8.9%	12.4%	13.9%	11.4%	5.9%	11.3%	16.3%	11.1%	<0.0001
Caesarean section (elective)	15.2%	12.1%	16.5%	15.7%	9.9%	10.1%	14.6%	14.6%	14.6%	<0.0001
Caesarean section (emergency)	11.3%	10.7%	12.0%	12.9%	9.2%	6.8%	14.7%	16.0%	11.9%	<0.0001
Stillbirth/1000 births	5.2/1 000	5.6/1 000	3.6/1 000	4.1/1 000	4.3/1 000	7.2/1 000	5.6/1 000	5.9/1 000	5.5/1 000	0.001
Neonatal death/1000 live births	1.9/1 000	2.3/1 000	1.8/1 000	1.6/1 000	2.2/1 000	2.5/1 000	1.7/1 000	2.5/1 000	1.9/1 000	0.57
Epidural usage	27.4%	23.4%	34.3%	27.7%	12.0%	13.2%	23.6%	33.8%	28.1%	<0.0001
No pain relief for labour	8.1%	11.9%	6.3%	5.2%	9.1%	13.9%	8.1%	3.5%	8.3%	<0.0001
Episiotomy	14.3%	10.6%	16.3%	23.8%	27.7%	9.2%	19.2%	31.5%	18.2%	<0.0001
Severe perineal trauma #	1.6%	1.4%	1.8%	1.6%	1.3%	1.4%	1.9%	1.4%	1.6%	0.10
Severe perineal trauma (primiparous women only)#	1.6%	1.6%	1.9%	1.9%	1.5%	1.2%	2.2%	1.2%	1.5%	0.04
Birth weight (mean SD)	3423 (580.4)	3449 (588.6)	3451 (547.7)	3350 (513.2)	3196 (502.2)	3393 (549.4)	3255 (545.5)	3157 (537.4)	3354 (566.9)	<0.0001
Birth weight <10th centile	9.4%	8.8%	8.3%	10.4%	15.8%	10.6%	12.9%	22.2%	11.9%	<0.0001
Birth weight <3rd centile	2.8%	2.7%	2.3%	2.6%	4.6%	2.9%	3.5%	8.1%	3.4%	<0.0001
5 minute Apgar <7	2.0%	2.1%	1.8%	1.5%	2.3%	2.1%	2.1%	2.1%	2.0%	<0001

*ANOVA not adjusted for congenital abnormalities.

#For severe perineal trauma and episiotomy caesarean sections were removed for analysis.

When comparing Australian-born women to the seven migrant groups where more than one percent of the birthing women were represented, there were some notable differences in demographics and medical and obstetric risk factors (Table 3). On average, women born in England were the oldest (33.8 years) while those born in Lebanon were the youngest (29.3 years). Indian women were more commonly primiparous (52.9%) and Lebanese women were least likely to be primiparous (27.7%). Women born in China had the lowest rates of smoking (0.5%) and New Zealand-born women had the highest rates of smoking (22.6%) - higher than for Australian born women. Lebanese-born women were the least likely to be a give birth in a private hospital (8.4%) compared to Australian-born women (26.0%). The lowest rates of hypertensive disease of pregnancy was amongst Vietnamese (2.5%) and Chinese women (2.8%), which was half that of Australian-born women (6.0%). Gestational diabetes was highest amongst women born in China (13.8%) and Vietnam (11.4%), around four times the rate seen in Australian-born women (3.1%). Women born in the Philippines had the highest rate of preterm birth (7.1%).

Rates of obstetric intervention also varied between the different migrant groups with women born in Vietnam having the highest rate of spontaneous labour (75.4%); those born in Lebanon had the highest rate of normal vaginal birth (76.8%); those born in India had the highest instrumental birth rate (16.3%), episiotomy (32%) and caesarean section rate (30.7%); and those born in England had the highest epidural rate (34.3%). Indian-born women had babies born with the lowest mean birth weights (3157 gms, SD 537.4), had the highest rate of babies with weights <10th centile (22.2%) and <3rd centile (8.1%). Vietnamese women had the highest number of babies born with Apgars<7 at five minutes (2.3%) and Lebanese born women had the highest rates of stillbirth (7.2/1000).

### Low risk primiparous and multiparous women

There were similar percentages of Australian and non-Australian-born women represented in the low risk primiparous group (43.2% vs 43.1%) but this was significantly different in the low risk multiparous group (43.7% vs 37.6%). Women born in England represented the lowest percentage mainly due to being on average older. Women born in Vietnam had the highest percentage (52.0%) represented in the low risk primiparous group with women born in Lebanon having the highest percentage (49.0%) represented in the low risk multiparous group.

### Low risk primiparous outcomes

When admission status (private or public hospital), obstetric interventions and outcomes for low risk primiparous women were examined, several differences were noted (Table 4). Indian-born women had the lowest spontaneous labour rate (46.4%), lowest normal vaginal birth rate (51.4%), highest instrumental birth rate (26.0%), highest episiotomy rate (40%) and caesarean section rate (22.7%); equal to that of women born in the Philippines. Women born in China also had a high instrumental birth rate (24.6%). While not statistically significant, Indian- born women also had the second highest stillbirth rate (2.6/1000) after New Zealand-born women (3.0/1000) and highest perinatal mortality rate (3.6/1000). The only obstetric indicator that Indian-born women demonstrated better outcomes on was severe perineal trauma with the lowest rate recorded in this group (0.9%), although the difference was not statistically significant due to low incidence of this outcome Table 4.

**Table 4:** Birth in a private hospital and obstetric outcomes for low risk primiparous Australian-born and migrant women, N=124,431

	**Australia**	**New Zealand**	**England**	**China**	**Vietnam**	**Lebanon**	**Philippines**	**India**	**Other**	**P**
	**n=88 437**	**n=2 663**	**n=2 361**	**n=2 915**	**n=3 123**	**n=1 727**	**n=1 689**	**n=1 959**	**n=19 557**	
Birth in a private hospital	27.4%	14.4%	21.1%	10.7%	10.1%	8.9%	12.1%	16.1%	19.2%	<0.0001
Spontaneous labour	52.1%	57.8%	55.1%	54.2%	59.3%	59.2%	58.3%	46.4%	53.8%	<0.0001
Normal vaginal delivery	58.4%	64.5%	57.7%	56.6%	65.2%	75.2%	56.7%	51.4%	59.5%	<0.0001
Assisted vaginal delivery	20.8%	18.5%	21.6%	24.6%	21.5%	15.0%	20.6%	26.0%	21.2%	<0.0001
Caesarean section (elective)	4.6%	3.0%	4.2%	3.6%	2.4%	1.4%	3.7%	3.8%	4.5%	<0.0001
Caesarean section (emergency)	16.0%	13.9%	16.4%	15.1%	10.9%	8.4%	19.0%	18.9%	14.8%	<0.0001
Stillbirth per 1000 births*	1.7/1000	3.0/1000	2.1/1000	1.4/1000	1.0/1000	2.3/1000	1.2/1000	2.6/1000	1.5/1000	0.59
Neonatal death per 1000 live births*	0.3/1000	0.0/1000	0.4/1000	0.7/1000	0.3/1000	1.2/1000	0.6/1000	1.0/1000	0.3/1000	0.56
5 minute Apgar <7	1.5%	1.3%	1.6%	0.9%	1.8%	1.6%	1.1%	1.5%	1.4%	0.21
Epidural usage	38.2%	33.5%	42.8%	35.8%	17.4%	22.1%	34.6%	41.2%	37.7%	<0.0001
No pain relief for labour	4.4%	6.2%	4.4%	2.6%	3.1%	5.5%	3.8%	1.9%	3.9%	<0.0001
Episiotomy #	24.9%	18.8%	22.7%	31.0%	41.8%	22.5%	29.8%	39.7%	29.1%	<0.0001
Severe perineal trauma)#	1.6	1.5	1.8	1.8	1.3%	1.1%	1.7%	0.9%	1.5%	0.34

*Following removal of those with congenital abnormalities.

#For severe perineal trauma and episiotomy caesarean sections were removed for analysis.

Lebanese-born women were least likely to give birth in a private hospital (8.6%), have a caesarean section (9.8%) and have an assisted vaginal delivery (15.0%). These women also had the highest normal vaginal birth rate (75.2%).

Women born in England had the highest rate of epidural usage (42.8%), while Vietnamese women had the highest rate of spontaneous labour (59.3%) and the lowest rate of epidural usage (17.4%).

### Low risk multiparous outcomes

When we examined admission status and obstetric interventions and outcomes for low risk multiparous women several differences and similarities to the low risk primiparous data were noted (Table 5). Australian-born women continued to have the highest rate of births in a private hospital (20.2%), with Lebanese women having the lowest rate (6.7%). Indian-born women still had the lowest rate of normal vaginal birth (70.8%), the highest rates of assisted vaginal delivery (7.7%), highest episiotomy rate (18%) and caesarean section (21.4%). Vietnamese-born multiparous women continued to have the highest rate of spontaneous labour (82.0%) and the lowest rate of epidural usage (4.6%). Women born in England had the lowest rate of spontaneous labour (60.8%) and the highest rate of epidural usage (20.9%) (Table 5).

**Table 5:** Birth in a private hospital and obstetric outcomes for low risk multiparous Australian-born and migrant women, N=169,409

	**Australia**	**New Zealand**	**England**	**China**	**Vietnam**	**Lebanon**	**Philippines**	**India**	**Other**	**p**
	**n=127 528**	**n=4 388**	**n=2 162**	**n=2 098**	**n=3 341**	**n=4 525**	**n=2 076**	**n=1 468**	**n=21 823**	
Birth in a private hospital	20.2%	7.1%	18.7%	8.2%	7.5%	6.7%	7.9%	12.9%	13.3%	<0.0001
Spontaneous labour	61.4%	71.1%	60.8%	74.7%	82.0%	73.0%	76.9%	64.4%	83.6%	<0.0001
Normal vaginal delivery	77.7%	83.2%	77.1%	79.9%	86.6%	88.5%	77.8%	70.8%	76.9%	<0.0001
Assisted vaginal delivery	3.8%	2.6%	3.9%	3.4%	3.9%	2.2%	4.5%	7.7%	3.8%	<0.0001
Caesarean section (elective)	13.9%	9.7%	14.8%	11.7%	6.4%	6.5%	11.2%	15.2%	11.2%	<0.0001
Caesarean section (emergency)	4.6%	4.5%	4.2%	5.0%	3.1%	2.8%	6.5%	6.2%	5.0%	<0.001
Stillbirth/1000 births*	1.2/1000	1.1/1000	0.5/1000	1.4/1000	1.5/1000	2.2/1000	0.5/1000	2.0/1000	1.6/1 000	0.38
Neonatal death/1000 livebirths*	0.2/1000	0.0/1000	0.0/1000	0.5/1000	0.6/1000	0.7/1000	0.0/1000	0.0/1000	0.3/1 000	0.43
5 minute Apgar <7	0.9%	0.7%	0.9%	0.6%	0.8%	0.8%	0.6%	0.8%	0.9%	0.70
Epidural usage	16.9%	12.5%	20.9%	12.0%	4.6%	7.7%	12.9%	18.9%	15.8%	<0.0001
No pain relief for labour	17.0%	25.1%	13.4%	14.1%	19.1%	23.6%	19.2%	10.5%	18.9%	<0.0001
Episiotomy #	6.6%	3.6%	8.6%	11.7%	15.3%	3.8%	9.0%	17.5%	8.4%	<0.0001
Severe perineal trauma#	1.5%	1.4%	1.6%	1.5%	1.2%	1.4%	1.9%	1.1%	1.4%	0.70

*Following removal of those with congenital abnormalities.

#For severe perineal trauma and episiotomy, caesarean sections were removed for analysis.

## Discussion

How country of birth may impact on childbearing women has been the subject of several international and national studies. In this study we examined eight years of all singleton births in NSW and using a established low risk primiparous and multiparous criteria to identify a low risk cohort [33,35], were able to show some significant disparities that deserve discussion and ongoing investigation.

Low risk Indian-born women appear to have the highest rates of obstetric intervention during birth even when they are identified as low risk. The rate of babies born with birth weights <10th centile in this group is also extremely high at 22.2%, more than twice the rate for Australian-born women (9.4%). The perinatal mortality rate for babies born to low risk Indian-born primiparous women was the highest of any group examined, though this was not statistically significant. Lebanese women also experienced similar perinatal death rates to Indian women and overall had the highest stillbirth rate. It is interesting to note that while not statistically significant the highest perinatal death rates occurred in populations with the highest obstetric intervention rates (Indian born women) and lowest obstetric intervention rates (Lebanese born women), challenging recent obstetric arguments that high rates of obstetric intervention improve perinatal outcomes [36].

It is possible that intrauterine growth varies with different ethnic backgrounds. For example, Wen et al. (1995) reported more rapid fetal growth early on in the third trimester and slower growth near or after term amongst women born in China compared to European women [29,37].

Studies have indicated that sociodemographic factors such as income may have an influence on birth weight [13] and these factors affect migrants to a greater extent. Shorter height, being underweight and smoking during pregnancy have higher prevalence in lower income groups [13]. Hayward et al. (2012) suggest height may indicate trans-generational aetiology for socioeconomic birth weight inequalities and/or that adult height influences social mobility. Adult height changes over generations and hence can be an indicator of multigenerational histories of conditions that facilitate growth [38]. However, misclassifying healthy but constitutionally small babies, may lead to unnecessary monitoring and interventions [29]. It is unclear whether small babies, for example, born to women with shorter height, are at increased risk of adverse outcomes and what could be done about it [13]. While we did not have access to data on height it is known that women born in India are of shorter stature than those born in Australia and this may be associated with the high rates of babies with birth weights <10th and <3rd centile identified in this group. A recent randomised controlled trial of caseload midwifery (one lead midwife providing antenatal, intrapartum and postnatal care) compared to standard models of care (involving many different caregivers) showed a halving in the incidence of low birth weight babies when women had continuity of care, along with substantial reductions in interventions, including a 22% reduction in the caesarean section rate [39]. There is potential for future studies to examine the impact of continuity of midwifery care on specific migrant groups such as women born in India, which to date has not been done.

The higher rate of stillbirth amongst Lebanese-born women needs to be explored further and may be linked to a higher rate of consanguinity. While we were able to control for congenital abnormalities there are certain cultural and religious beliefs that lead to low autopsy rates and hence identification of congenital abnormalities in some migrant groups.

Studies undertaken in Lebanon have found rates of consanguinity around 35.5%, of which 31.6% were first cousins [40] and this rate is higher in Muslim communities and where education level is low [40]. In some Middle Eastern populations this practice has been reported as high as 50% [41]. A study undertaken in Australia examining referrals to a genetic counseling clinic in a NSW hospital found a strong influence of consanguinity amongst Middle Eastern populations on autosomal recessive genetic diseases [42]. A study undertaken in the 1980s in NSW showed a prevalence of congenital abnormalities, perinatal mortality and morbidity of almost three times higher amongst children born to Lebanese born women [43] and that 35.8% of marriages in the community were consanguineous [44], similar to the rate found by Barbour and Salameh in Lebanon in 2009. What the rate of consanguinous marriages is today in Australia is however unknown.

The rates of smoking varied between the different groups. Australian-born women had a much higher rate (18.3%) than non-Australian-born women (6.1%). However, the New Zealand-born women had the highest rate of all the groups (22.6%) and Vietnamese women had the lowest rates (0.5%). In the low risk primiparous group women who smoked had been excluded yet the New Zealand-born women had the highest stillbirth rate (3.0/1000), though this was not statistically significant. While we were unable to determine what proportion of these women were Maori or Pacific Islander, the rate of stillbirth in these populations is higher than for other women in New Zealand [45].

The high incidence of gestational diabetes, particularly amongst Chinese-born and Vietnamese born women, is supported by other studies [46]. Gestational diabetes carries risk such as higher fetal weight and birth complications for the baby [47] and an increased risk of the woman developing Type-2 diabetes [48].

There were significant differences between groups for the rates of teenage pregnancies, with the highest rate being in the Australian-born group (5.1%) and lowest rate amongst Indian-born women (0.1%). Non-Australian-born women were much more likely to be over 35 years of age (26.2%) compared to Australian-born women (17.8%), though women born in India had the lowest rate (13.2%). With low rates of smoking, teenage pregnancy and older age, this makes the finding of high rates of babies with birth weights <3rd and <10th centile even more curious in this population.

There were also significant differences in numbers of women giving birth in a private hospital under the care of a private obstetrician. Overall, Australian-born women were significantly more likely to give birth in a private hospital compared to non-Australian-born women (26% vs 17.7%). A previous study demonstrated high rates of obstetric intervention in women who gave birth in private hospitals compared to public hospitals [33]. While private insurance coverage is an indicator of social advantage and all the perinatal benefits that come from this, we found equal or better outcomes amongst some of the population groups with low levels of birth in private hospitals, showing this is a complex area requiring further research.

There are significant advantages of using population-based datasets such as the MDC, including the size of the dataset, the guaranteed accuracy of a validated dataset [49,50] and the anonymous nature of the results therein.

The limitations are the limited number of variables that are included and the scarcity of specific information on potential confounders. We also cannot be sure that women’s country of birth always relates to their ethnicity. There are many variables not available in routinely collected data that impact on outcomes for migrant women and their babies. These include, but are not limited to, English language fluency, migration from an English speaking country, length of residence, refugee background and factors associated with care such as discrimination and culturally insensitive care [51]. We were unable to control for socio economic status and this could influence results, as low socioeconomic status has been identified as more common in migrant families [51]. We were also unable to control for refugee and asylum seeker status and these women in particular are at increased risk of poorer outcomes due to a history of gender based violence and poor nutrition [52]. Likewise we could not tell how long the women had been in Australia and there is some evidence of the healthy migrant effect where newly arrived individuals show better health outcomes initially but these worsen over time [53]. Lack of migrant relevant data for assessing maternal and perinatal outcomes continues to limit more in-depth understandings in Australia and many other countries pursing this important research agenda [51].

## Conclusion

The results of this study suggest there are significant differences in risk profiles, rates of obstetric interventions and maternal and neonatal outcomes between Australian-born and women born overseas and these differences are seen overall and in low risk populations. With Indian-born women now the leading migrant group coming into Australia and having the lowest normal birth rate, along with high rates of babies with birth weights <10th and <3rd centile, attention needs to be focused on identifying effective models of care that might improve these outcomes for this population.

## Competing interests

The authors declare that they have no competing interests.

## Authors’ contributions

HD designed the study, worked on analysis, and wrote the paper. VS helped design the study and assisted with writing of the paper. CLD assisted with advice interpreting results and writing of the paper. CT helped design the study, led the analysis and also assisted with writing the paper, in particular the method. All authors read and approved the final manuscript.

## Pre-publication history

The pre-publication history for this paper can be accessed here:

http://www.biomedcentral.com/1471-2393/13/100/prepub

## References

[B1] Australian Government Citizenship DoIa The people of Australia: Australia’s multicultural policy 2011 Canberra: Australian Government

[B2] Duttagupta I With Australian govt’s incentives, Indian immigrants are moving to regional & rural centers in the country The Economic Times vol http://articles.economictimes.indiatimes.com/2012-07-29/news/32907686_1_source-of-permanent-migrants-permanent-migration-programme-immigration-and-citizenship; July 29th 201222974314

[B3] Malin M Gissler M Maternal care and birth outcomes among ethnic minority women in Finland BMC Publ Health 2009 9 84 10.1186/1471-2458-1189-1184 PMC2674879 19298682

[B4] Waterstone M Bewley S Wolfe C Incidents and predictors of severe obstetric morbidity: case–control study BMJ 2001 322 5 May 1089 1094 11337436 10.1136/bmj.322.7294.1089 PMC31259

[B5] Stirbu I Kunst A Bos V Mackenbach J Differences in avoidable mortality between migrants and the native Dutch in the Netherlands BMC Publ Health 2006 6 78 10.1186/1471-2458-6-78 PMC1435889 16566833

[B6] Taylor F Ko R Pan M Kramer A Prenatal and reproductive health care Immigrant women’s health Problems and solutions. edn 1999 San Francisco: Jossey-Bass Publishers

[B7] Lalchandani S MacQuillan K Sheil O Obstetric profiles and pregnancy outcomes of immigrant women with refugee status Irish Medical Journal 2001 94 3 79 80 11354688

[B8] Essén B Johnsdotter S Hovelius B Gudmundsson S Sjöberg NO Friedman J Ostergren PO Qualitative study of pregnancy and childbirth experiences in Somalian women residents in Swe- den British Journal of Obstetrics and Gynecology 2000 107 1507–12 10.1111/j.1471-0528.2000.tb11676.x 11192108

[B9] Vangen S Stoltenberg C Krondal A Magnus P Stray-Pedersen B Cesarean section among immigrant in Norway Acta Obstetetrica Gynecologica Scandinavia 1999 79 553 558 10929954

[B10] Gissler M Alexander S Macfarlane A Small R Stray-Pedersen B Zeit- lin J Zimbeck M Gagnon AftRcROaMAIRC Stillbirths and infant deaths among migrants in industrialised countries Acta Obstetr Gynaecol Scand 2009 88 134 148 10.1080/00016340802603805 19096947

[B11] Troe E Bos V Deerenberg I Mackenbach J Joung I Ethnic differences in total and cause-specific infant mortality in The Netherlands. 2006, 20:140–7 Paediatric Perinat Epidemio 2006 20 140 147 10.1111/j.1365-3016.2006.00699.x 16466432

[B12] Richardus J Graafmans W Verloove-Vanhorick S Mackenbach JEIAP EuroNatal Working Group: Differences in perinatal mortality and suboptimal care between 10 European regions: results of an international audit BJOG 2003 110 97 105 10.1046/j.1471-0528.2003.02053.x 12618151

[B13] Hayward I Malcoe LH Cleathero LA Janssen PA Lanphear BP Haynes MV Mattman A Pampalon R Venners SA Investigating maternal risk factors as potential targets of intervention to reduce socioeconomic inequality in small for gestational age: a population-based study BMC Publ Health 2012 12 333 http://www.biomedcentral.com/1471-2458/12/333 10.1186/1471-2458-12-333 PMC3518824 22569183

[B14] Agyemang C Vrijkotte T Droomers M van der Wal M Bonsel G Stronks K The effect of neighbourhood income and deprivation on pregnancy outcomes in Amsterdam, The Netherlands J Epidemiol Community Health 2009 63 755 760 10.1136/jech.2008.080408 19679715

[B15] Luo Z Kierans WJ Wilkins R Liston RM Mohamed JMSK Disparities in birth outcomes by neighbourhood income: temporal trends in rural and urban areas, British Columbia Epidemiology 2004 15 6 679 686 10.1097/01.ede.0000142149.34095.88 15475716

[B16] Gillman M Rich-Edwards J The fetal origins of adult disease: from sceptic to convert Paediatr Perinat Epidemiol 2000 14 192 193 10.1046/j.1365-3016.2000.00265.x 10949210

[B17] Indredavik M Vik T Heyerdahl S Kulseng S Fayers P Brubakk AM Psychiatric symptoms and disorders in adolescents with low birthweight Child Care Health Dev 2005 31 1 121 125

[B18] Merry L Small R Blondel B Gagnon AJ International migration and caesarean birth: a systematic review and meta-analysis BMC Pregnancy and Childbirth 2013 27 http://www.biomedcentral.com/1471-2393/13/27 23360183 10.1186/1471-2393-13-27 PMC3621213

[B19] Merten S Wyss C Ackermann-Liebrich U Caesarean sections and breastfeeding initiation among migrants in Switzerland Int J Public Health 2007 52 210 222 10.1007/s00038-007-6035-8 18030953

[B20] Ell K Castaneda I Health seeking behaviour Handbook of immigrant health. edn 1998 New York: Plenum 125 143

[B21] Blais R Maiga A Do ethnic groups use health services like the majority of the population? A study from Québec, Canada Social Science and Medicine 1999 48 1237 1245 10.1016/S0277-9536(98)00423-7 10220022

[B22] Morris S Sutton MHG Inequity and inequality in the use of health care in England: an empirical investigation Social Science and Medicine 2005 60 1251 1266 10.1016/j.socscimed.2004.07.016 15626522

[B23] Adamson J Ben-Shlomo Y Chaturvedi NJD Ethnicity, socio-economic position and gender – do they affect reported health-care seeking behaviour? Social Science & Medi- cine 2003, 57:895–904 Social Science and Medicine 2003 57 895 904 10.1016/S0277-9536(02)00458-6 12850114

[B24] van Ryn M Fu SS Paved with good intentions: do public health and human service providers contribute to racial/ethnic dis- parities in health? American Journal of Public Health 2003, 93:9–36 Am J Public Health 2003 93 9 36 10.2105/ajph.93.2.248 PMC1447725 12554578

[B25] Delvaux T Buekens P Godin I Boutsen M Barriers to prenatal care in Europe American Journal of Preventive Medicine 2001 21 1 52 59 10.1016/S0749-3797(01)00315-4 11418258

[B26] Alderliesten M Vrijkotte T Wal M Bonsel G Late start of antenatal care among ethnic minorities in a large cohort of pregnant women BJOG 2007 114 1232 1239 10.1111/j.1471-0528.2007.01438.x 17655734

[B27] Schmied V Olley H Burns E Duff M Dennis C-L Dahlen HG Contradictions and conflict: A meta-ethnographic study of migrant women’s experiences of breastfeeding in a new country BMC Pregnancy and Childbirth 2012 12 163 http://www.biomedcentral.com/1471-2393/12/163 10.1186/1471-2393-12-163 PMC3546887 23270315

[B28] Auger N Luo ZC Platt RWMD Do mother’s education and foreign born status interact to influence birth outcomes? Clarifying the epidemiological paradox and the healthy migrant effect J Epidemiol Community Health 2008 62 402 409 10.1136/jech.2007.064535 18413452

[B29] Janssen PA Thiessen P Klein M Whitfield MF MacNab YC Cullis-Kuhl SC Standards for the measurement of birth weight, length and head circumference at term in neonates of European, Chinese and South Asian ancestry Open Medicine 2007 1 2 E74 E88 20101298 PMC2802014

[B30] Small R Yelland J Lumley J Brown S Liamputtong P Immigrant women’s views about care during labor and birth: an Australian study of Vietnamese, Turkish, and Filipino women Birth 2002 29 266 277 10.1046/j.1523-536X.2002.00201.x 12431266

[B31] Small R Rice P Yelland J Lumley J Mothers in a new country: the role of culture and communication in Vietnamese, Turkish and Filipino women’s experiences of giving birth in Australia Women Health 1999 28 77 101 10.1300/J013v28n03_06 10374809

[B32] ABS Standard Australian Classification of Countries (SACC) 2011 Edited by http://www.abs.gov.au/AUSSTATS/abs@.nsf/DetailsPage/1269.02011?OpenDocument

[B33] Dahlen HG Tracy S Tracy M Bisits A Brown C Thornton C Rates of obstetric intervention among low-risk women giving birth in private and public hospitals in NSW: a population-based descriptive study BMJ Open 2012 2 e001723 10.1136/bmjopen-2012-001723 22964120 PMC3467614

[B34] Roberts CL Tracy S Peat B Rates of obstetric intervention among private and public patients in Australia: population based descriptive study British Medical Journal 2000 312 137 141 10894690 10.1136/bmj.321.7254.137 PMC27430

[B35] Roberts CL Tracey S Peat B Rates for obstetric intervention among private and public patients in Australia: population based descriptive study British Medical Journal 2000 321 7254 137 141 10.1136/bmj.321.7254.137 10894690 PMC27430

[B36] Robson SJ Laws P Sullivan EA Adverse outcomes of labour in public and private hospitals in Australia: a population based descriptive study The Medical Journal of Australia 2009 190 9 474 477 19413516 10.5694/j.1326-5377.2009.tb02543.x

[B37] Wen S Kramer M Usher R Comparison of birth weight distributions between Chinese and Caucasian infants Am J Epidemiol 1995 141 12 1177 1187 7771456 10.1093/oxfordjournals.aje.a117391

[B38] Wells JC Maternal capital and the metabolic ghetto: An evolutionary perspective on the transgenerational basis of health inequalities Am J Hum Bio 2010 22 1 1 17 10.1002/ajhb.20994 19844897

[B39] McLachlan HL Forster DA Davey MA Farrell T Gold L Biro MA Albers L Flood M Oats J Waldenstrom U Effects of continuity of care by a primary midwife (caseload midwifery) on caesarean section rates in women of low obstetric risk: the COSMOS randomised controlled trial BJOG 2012 10.111/j.1471-0528.2012.03446.x 22830446

[B40] Barbour B Salameh P Consanguinity in Lebanon: prevalence, distribution and determinants J Biosoc Sci 2009 14 4 505 517 19175949 10.1017/S0021932009003290

[B41] Saadallah AA Rashed MS Newborn screening: experiences in the Middle East and North Africa Journal of Inherited and Metabolic Diseases 2007 30 482 489 10.1007/s10545-007-0660-5 17701444

[B42] Nelson J Smith M Bittles AH Consanguineous marriage and its clinical consequences in migrants to Australia Cinical Genetics 1997 52 142 146 10.1111/j.1399-0004.1997.tb02534.x 9377802

[B43] de costa C Congenital abnormalities and consanguinity Medical Journal of Australia 1986 144 721 722 3724606 10.5694/j.1326-5377.1986.tb113710.x

[B44] de costa C Pregnancy outcomes in Lebanese born women in Western Sydney Medical Journal of Australia 1988 149 457 460 3185340 10.5694/j.1326-5377.1988.tb120728.x

[B45] Ekeroma AJ Craig ED Stewart AW Mantell CD Mitchell EA Ethnicity and birth oiutcome: New Zealand trends 1980–2001: Part three pregnancy outcomes for Pacific women ANZJOG 2004 44 6 541 544 15598293 10.1111/j.1479-828X.2004.00311.x

[B46] Carolan M Gestational diabetes mellitus among women born in South East Asia: A review of the evidence Midwifery 2013 Online Feb 2013 10.1016/j.midw.2012.09.003 23415355

[B47] Crowther CA Hiller JE Moss JR McPhee AJ Jeffries WS Robinson JS Australian Carbohydrate Intolerance Study in Pregnant Women (ACHOIS) Trial Group Effect of treatment of gestational diabetes mellitus on pregnancy outcomes New England Journal of Medicine 2005 352 24 2477 2486 10.1056/NEJMoa042973 15951574

[B48] Lee A Hiscock R Wein P Walker S Permezel M Gestational Diabetes Mellitus: Clinical Predictors and Long-Term Risk of Developing Type 2 Diabetes. A retrospective cohort study using survival analysis Diabetes Care 2007 30 4 878 883 10.2337/dc06-1816 17392549

[B49] Taylor L Travis S Pym M Olive E Henderson-Smart D How useful are hospital morbidity data for monitoring conditions occurring in the perinatal period? Australian & New Zealand Journal of Obstetrics & Gynaecology 2005 45 36 41 10.1111/j.1479-828X.2005.00339.x 15730363

[B50] Roberts C Bell J Ford J Morris J Monitoring the quality of maternity care: how well are labour and delivery events reported in population health data? Paediatirc and Perinatal Epidemiology 2008 23 144 152 10.1111/j.1365-3016.2008.00980.x 19159400

[B51] Gagnon AJ Zimbeck M Zeitlin J Collaboration ROAM Alexander S Blondel B Buitendijk S Desmeules M Di Lallo D Gagnon A Gissler M Glazier R Heaman M Korfker D Macfarlane A Ng E Roth C Small R Stewart D Stray-Pederson B Urquia M Vangen S Zeitlin J Zimbeck M Migration to western industrialised countries and perinatal health: a systematic review Soc Sci Med 2009 69 6 934 946 10.1016/j.socscimed.2009.06.027 19664869

[B52] Krause SK Jones RK Purdin SJ Programmatic responses to refugees’ reproductive health needs International Family Planning Perspectives 2000 26 181 187 10.2307/2648256

[B53] Gushulak B Healthier on arrival? Further insight into the “healthy immigrant effect” Canadian Medical Association Journal 2007 176 1439 1440 10.1503/cmaj.070395 17485696 PMC1863517

